# The hierarchical and functional connectivity of higher-order cognitive mechanisms: neurorobotic model to investigate the stability and flexibility of working memory

**DOI:** 10.3389/fnbot.2013.00002

**Published:** 2013-02-18

**Authors:** Fady Alnajjar, Yuichi Yamashita, Jun Tani

**Affiliations:** ^1^RIKEN Brain Science InstituteWako, Saitama, Japan; ^2^JST, ERATO, Okanoya Emotional Information ProjectWako, Saitama, Japan; ^3^Department of Electrical Engineering, Korea Advanced Institute of Science and TechnologyYuseong-gu, Daejeon, Republic of Korea

**Keywords:** higher-order cognitive tasks, brain modeling, cognitive branching task, multi-timescale recurrent neural network, working memory, frontal lobe function

## Abstract

Higher-order cognitive mechanisms (HOCM), such as planning, cognitive branching, switching, etc., are known to be the outcomes of a unique neural organizations and dynamics between various regions of the frontal lobe. Although some recent anatomical and neuroimaging studies have shed light on the architecture underlying the formation of such mechanisms, the neural dynamics and the pathways in and between the frontal lobe to form and/or to tune the stability level of its working memory remain controversial. A model to clarify this aspect is therefore required. In this study, we propose a simple neurocomputational model that suggests the basic concept of how HOCM, including the cognitive branching and switching in particular, may mechanistically emerge from time-based neural interactions. The proposed model is constructed such that its functional and structural hierarchy mimics, to a certain degree, the biological hierarchy that is believed to exist between local regions in the frontal lobe. Thus, the hierarchy is attained not only by the force of the layout architecture of the neural connections but also through distinct types of neurons, each with different time properties. To validate the model, cognitive branching and switching tasks were simulated in a physical humanoid robot driven by the model. Results reveal that separation between the lower and the higher-level neurons in such a model is an essential factor to form an appropriate working memory to handle cognitive branching and switching. The analyses of the obtained result also illustrates that the breadth of this separation is important to determine the characteristics of the resulting memory, either static memory or dynamic memory. This work can be considered as a joint research between synthetic and empirical studies, which can open an alternative research area for better understanding of brain mechanisms.

## Introduction

Higher-order cognitive mechanisms (HOCM) refer to the ability to coordinate thought and action through working memory toward obtaining a specific goal. Its major functions are to perform complex sequences of behaviors and give priority to goals and sub-goals. HOCM are known to have a direct association with the size and the complexity of the prefrontal cortex (PFC) (Grafman, 1995; Koechlin et al., 1999), and thus are characteristic of higher primate mammalians, reaching a peak in humans (Miller and Wallis, 2009). PFC damage adversely affects the performance of these mechanisms (Elliott et al., 1996; Channon and Green, 1999; Walsh et al., 2009). Given this background, many researchers have focused studying HOCMs, such as planning, cognitive branching, switching, etc. (Grafman, 1995; Elliott et al., 1996; Channon and Green, 1999; Koechlin et al., 1999; Miller and Wallis, 2009; Walsh et al., 2009).

Various anatomical and neuroimaging studies have attempted to outline the basics behind the emergence of HOCM, reporting hypotheses on the architecture and the neural pathways surrounding the anterior frontal lobe (Braver and Bongiolatti, 2002; Koechlin and Hyafil, 2007; Hagmann et al., 2008; Badre and D'Esposito, 2009). At the neuroimaging level, for instance, some studies focused on the most anterior lateral regions of the frontal lobe, namely the frontopolar cortex (FPC), the mid-dorsolateral PFC (Mid-PFC), and the dorsal premotor cortex (Pre-PMD), in efforts to understand the specific nature of the mechanism underlying the activity related to sub-goal processing and primary task retrieval from the working memory (Konishi et al., 2000; Lepage et al., 2000; McDermott et al., 2000). At the anatomical level, Bongiolatti (Braver and Bongiolatti, 2002) argued that the FPC, in particular, is directly engaged in cognitive branching processes due to its distinctive position. Despite progress in anatomical and neuroimaging research, there are still some limitations. The underlying neural dynamics in and between various regions in the frontal lobe to self-organize a temporal working memory to obtain HOCM, as well as, to tune its stability and flexibility, remain a fundamental challenge in neuroscience. A model to suggest a general perception of this process is, therefore, required.

Recently, neurocomputational research has been considered as an alternative/support approach to explore neural dynamics in the brain (Botvinick and Plaut, 2006; Johnson et al., 2009; Maniadakisa et al., 2012; Yamashita and Tani, 2012). Such studies attempt to establish detailed links between biology and cognition in a manner that is consistent with established neural information processing principles from anatomical and neuroimaging studies. Neurocomputational modeling seeks to describe functional principles of how the simulated neural system in the brain operates through a relatively comprehensible set of equations, and has been a powerful tool for clarifying several mechanisms of neural systems (Maniadakisa et al., 2012; Yamashita and Tani, 2012).

**Figure 1:**
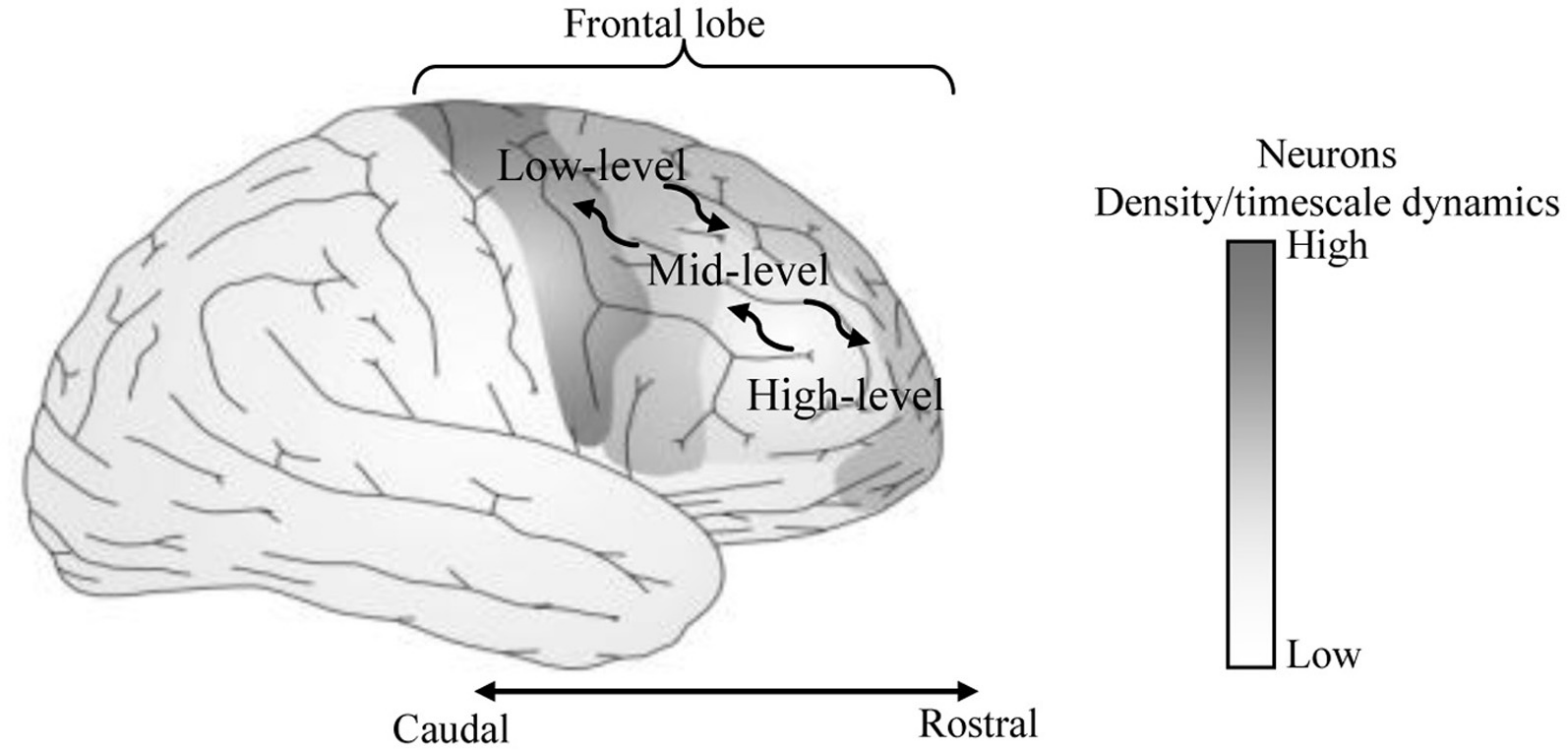
**Anatomical sites of area of our focus on the human brain.** The most anterior regions show lower number of neurons and slower neural timescale dynamics than the most posterior regions.

**Figure 2:**
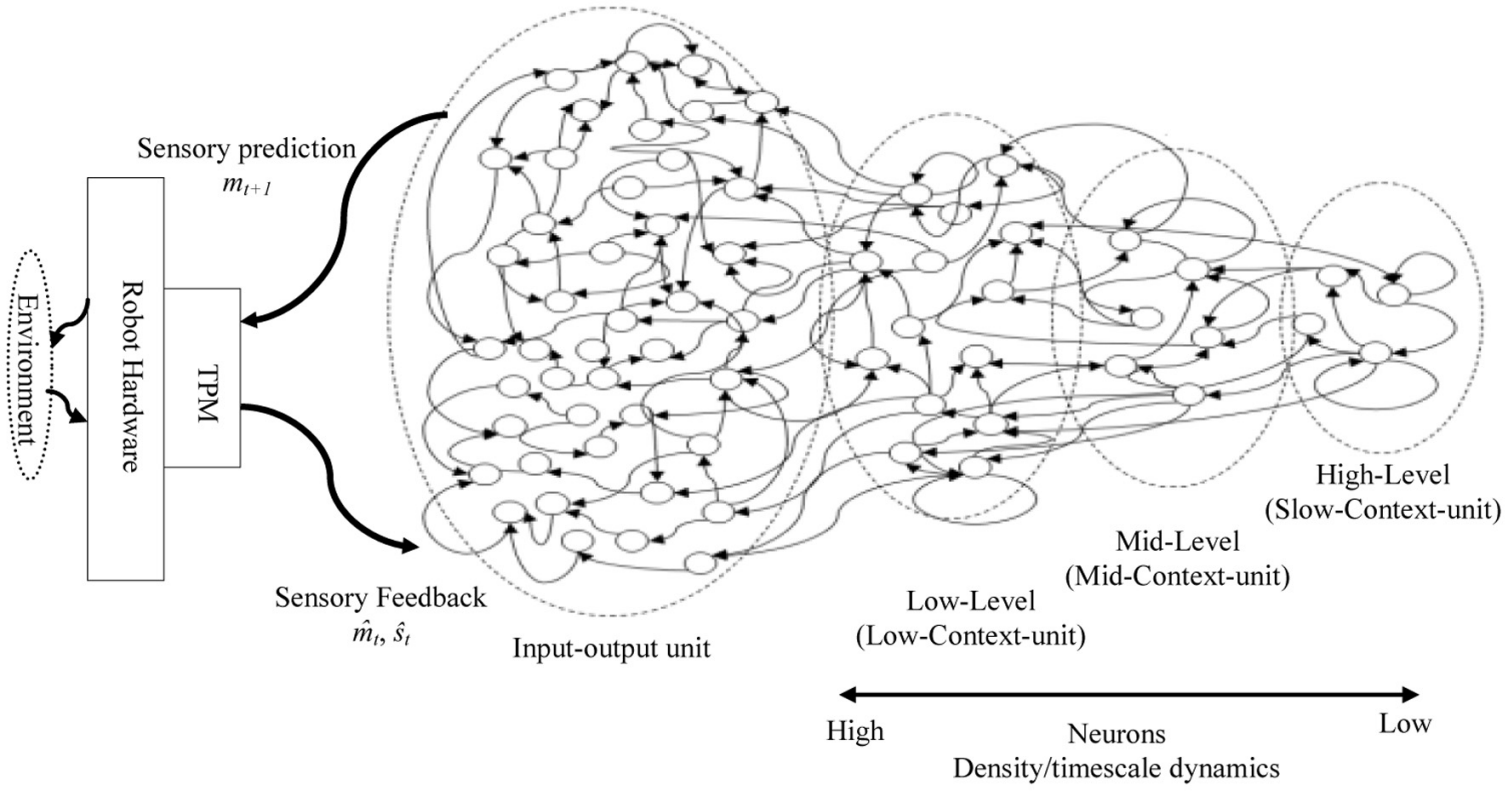
**A sample of the proposed MTRNN model.** The basic layout of the model inspired by Figure 1. In the model, neurons in the slow context unit have lower number and slower timescale dynamics than the neurons in the fast context unit. Inputs and outputs of the model are processed using topology preserving maps (TPM) (details are in the “Model Overview” section).

A hierarchical Multi-Timescale Recurrent Neural Network (MTRNN) modeling, adopted in this study, has recently been considered as a possible neurocomputational model that simulates, to some extent, the brain activities (Yamashita and Tani, 2008, 2012). MTRNN has ability to simulate the functional hierarchy of the brain through self-organization that is not only based on the spatial connection between neurons, but also on distinct types of neuron activities, each with distinct time properties; see Figures 1, 2. By such varied neuron activities, continuous sequences of any set of behavior are segmented into reusable primitives, which in turn are flexibly integrated into diverse sequential behaviors. The biological approval of such a type of hierarchy has been discussed in detail by Badre (Badre and D'Esposito, 2009), who suggested that levels of abstraction might gradually reduce along the rostro-caudal axis in the frontal cortex of the monkey and human brain. Others have also argued that the rostral part is more integrative in processing information than the caudal part due to its slower timescale dynamics, which results in the formation of functional hierarchy in the frontal cortex. Thus, neurons in higher levels process abstract action goals in slow dynamics, and those close to motor regions process in faster dynamics concrete information about actions that is closer to the actual motor output; see Figure 1.

In this study, we propose a neurocomputational model (MTRNN) that may suggest the basic perception of how a working memory may mechanistically be self-structured through neural interactions to accomplish HOCM, namely: (1) cognitive branching tasks: in which a delay to the execution of the original task occurs until the completion of a subordinate task; and (2) switching: in which the original task is abandoned for a new one. A physical humanoid robot implemented with the proposed model was considered for examining the model validity in real-time environmental interactions. We examined two possible MTRNN models to simulate the tasks, and discussed in detail the neural dynamics and memory formation of the superior model. The biological plausibility and the limitations of the resulting model are also addressed in this study. We believe that this joint research between synthetic and the empirical studies can open a new avenue for better understanding of brain mechanisms.

## Methods

### Task design

A miniature humanoid robot, produced by Sony Corporation, was used in the role of a physical body interacting with the actual environment. Instead of relying only on a simulation experiment, in this study, the robotics experiment is important when considering the idea of the embodied mind by Varela et al. (1991), who explained that cognitive functions of neural systems emerge not only in the brain, but also in dynamic interactions between a physical body and its environment. This idea is also related to the so-called “synthetic approach” to neuroscience (or “robotic neuroscience”), an approach that mainly aims to extract essential mechanisms of neural systems using a variety of neuro-cognitive robotics experiments (Doya, 2001).

**Figure 3:**
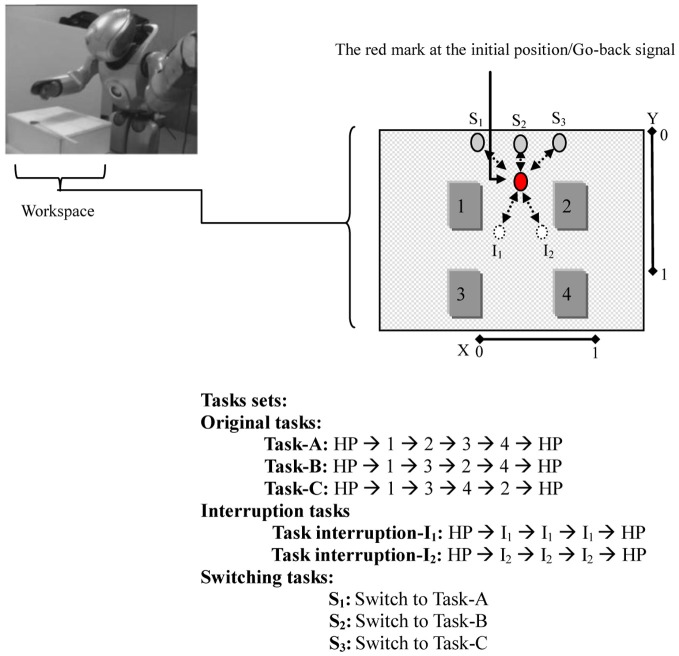
**The Robot (a miniature humanoid robot produced by Sony Corporation), the workspace, and the tasks sets.** In the workspace: the movable red mark is first located at the initial position. The red mark at this position stimulates the robot to execute the original task. The white circles/(gray circles) represent the interruption/(switching) positions. Moving the red mark to one of these positions stimulates the robot to execute the corresponding task. The dotted arrows indicate the paths of the red mark. In the tasks sets: home-position (HP) refers to the robot's arm in its initial position. In one Task-A set, for instance, the robot starting its arm from HP, moves to dial 1 → 2 → 3 → 4, and then ends the task by returning its arm to HP.

The robot was fixed to a stand. The selected tasks involve movements of the head and the right arm of the robot. The arm moves with four degrees of freedom assigned to ${\hat{m}}_{t}$ (four-dimensional vectors representing the angles of the arm joints), and the robot head's motor moves with two degrees of freedom assigned to ${\hat{s}}_{t}$ (two-dimensional vectors *x* and *y* representing the stimulus status: a red mark position, see Figure 3). The joints of the robot have rotation ranges that were scaled into values between [0.0 ~ 1.0]. Encoder values of these arm joint sensors were received as the current proprioceptive sensory feedback and sent to the network. A vision system mounted on the robot's head was programmed to locate the red mark on the workspace. The direction of the robot's head, indicated by encoder values of the two neck joints, expressed the red mark position in the visual field relative to the robot. This relative location of the red mark was treated as visual input ${\hat{s}}_{t}$ to the system to observe the current stimulus status and thus trigger the corresponding task; see Figure 3.

For the workspace, a workbench was set up in front of the robot, as in Figure 3. A sheet of white paper, which shows four numbers, interruptions, and switching positions, and a moveable red mark were placed on the workbench to conduct the experiment. The robot's task was to learn to reproduce cognitive branching and switching. For the cognitive branching: (1) the robot starts an original task that is determined by the experimenter (task-A, task-B, or task-C). In this task, as long as the red mark is located on the initial position, the robot should keep dialing the assigned task set by clicking on the numbers that the task presents using its right arm index finger. (2) When an external stimulus appears before the robot [i.e., the red mark is placed by the experimenter to one of the interruption positions (I_1_, I_2_)], the robot should suspend working on dialing the original task sets and execute the interruption subtask set (clicking directly on the red mark on its interruption position: I_1_ or I_2_. (3) When the red mark is returned, by the experimenter, to its initial position, which is a Go-Back signal, the robot should resume its outstanding original task starting with its arm in its home-position (HP). For the task switching, as done after starting the original task, if the red mark is temporarily (approximately two seconds) placed at one of the switching positions, S_1_, S_2_, or S_3_, and returned to the initial position, the robot should switch from the current original task to either task-A, task-B, or task-C, respectively.

### Model overview

The main components of the current model are borrowed from a continuous time recurrent neural network (CTRNN) (Elman, 1990). CTRNN is a type of RNN that implements some features of biological neurons, thus the activities of neurons are determined not only by the current synaptic inputs but also by the history of neural states. Due to these characteristics, CTRNN can reproduce complex dynamics as well as continuous sensorimotor sequences.

To construct a hierarchy structure of CTRNN that simulates the biological assumptions in Figure 1, we adopted the model of the MTRNN presented in Figure 2. The functional hierarchy in MTRNN is made possible through the use of various distinct types of neurons, each with different timescale properties. Thus, the time constant τ in (1), represents the activity speed of the corresponding neuron (Hertz et al., 1991; Yamashita and Tani, 2008):

(1) $$\tau_{i}{\dot{u}}_{i,t}=\;-u_{i,t}+\sum_{j}w_{ij}x_{j,t}$$

where *u*_*i,t*_ is the membrane potential, *x*_*i,t*_ is the neural state of the *i*th unit, and *w*_*ij*_ is the synaptic weight from the *j*th unit to the *i*th unit. The second-term of the equation corresponds to synaptic inputs to the *i*th unit. The time constant τ is defined as the decay rate of the unit's membrane potential, analogous to the leak current of membrane potential in real neurons. When the τ value is large, the activation of the unit changes slowly, because the internal state potential is strongly affected by the history of the unit's potential. On the other hand, when the τ-value of a unit is small, the effect of the history of the unit's potential is also small, and thus it is possible for activation of the unit to change quickly.

The network that is used in the current model consists of input-output and a context unit. To test various model layouts, the context unit is divided into two or three levels based on the value of the time constant τ, as well as, the number of neurons in each unit. An example of a general layout connection of a three-level context model is shown in Figure 2. Table 1 presents the detailed specifications for each model. The settings of neuron initial states are self-organized through the learning process (Nishimoto et al., 2008), and thus the initial values that correspond to the same behavior are close to each other in the state space of the initial values.

**Table 1:** **Specifications of the MTRNN models**.

**Specifications**	**Two-level model**	**Three-level model**
Structure	Input/output unit and a two-level context unit: fast context unit and slow context unit	Input/output unit and a three-level context unit: fast context unit, mid-context unit and slow context unit
Number of neurons in the input-output unit/(τ)	61/(τ = 2)	61/(τ = 2)
Number of neurons in the fast context unit/(τ)	16/(τ = 5)	14/(τ = 3)
Number of neurons in the mid-context unit/(τ)	0	10/(τ = 5)
Number of neurons in the slow context unit/(τ)	14/(τ = 50)	6/(τ = 50)
Connection among units	As in Figure 4, neurons in each unit are connected to themselves and only to the neurons in its direct neighbors units, as shown by the arrows in the figure.	As in Figure 6, neurons in each unit are connected to themselves and only to the neurons in its direct neighbors units, as shown by the arrows in the figure.

In the current study, parameters of time constants are set by the experimenters on a trial and error base. In Yamashita and Tani (2008), it was shown that time constant differences do not result in worse performance. In Paine and Tani (2005), it was shown that optimization of the time constants by an evolutionary algorithm applied to the learning of goal-directed navigation tasks results in the generation of similar time constant structures as those applied here, i.e., fast time constants in a lower-level for encoding primitive behaviors such as turning left/right and a straight corridor and slow time constants in higher-level for encoding sequences of primitives. Our future study, however, will focus on how the time constant parameters can be self-determined by utilizing the back-propagation error deliberated at each neural unit.

#### Input-output encoding to MTRNN

Inputs to the system were sparsely encoded in the form of a population coding using conventional topology preserving maps (TPM) (Kohonen, 1996), Figure 2. One map corresponds to proprioception and the other map to vision. The TPM is a type of neural network that produces a discretized representation of the input space of training samples. The characteristic feature of the TPM is that it preserves topological properties of the input space. This sparse encoding of sensorimotor trajectories reduces the overlaps of sensorimotor sequences. The size of the TPMs was 36 (6 × 6) for proprioception and 25 (5 × 5) for vision, respectively. The 6-dimensional proprioceptive and visual inputs were therefore transformed into 61-dimensional sparsely encoded vectors.

In this study, TPMs were trained in advance of MTRNN training using a conventional unsupervised learning algorithm (Kohonen, 1996). Samples for training of the TPMs included all the recorded teaching sensorimotor sequences of all the tasks in various combinations (a total of 16 recorded combinations designed arbitrary, e.g., combination 1: *Task−A* × 3 → *I*_2_ × 3 → *Task−A* × 3 (which means a three sets of Task-A followed by a three sets of *I*_2_, and then followed again by three sets of Task-A); combination 2: *Task−A* × 6 → *I*_2_ × 2 → *Task−A* × 2, etc.). In the training stage of the TPM, data were sampled randomly, and training for both proprioception and vision were carried out over a total of 3 × 10^6^ epochs.

Reference vectors of the TPM are described as follows:

(2) $$k_{i}=\left\{k_{i,1},k_{i,2},\ldots ,k_{i,l(i)}\right\}$$

where *l(i)* is dimension of the reference vector corresponding to the sample vectors of proprioception *m*_*t*_ or vision *s*_*t*_. Thus, *l(i)* is determined as follows: if *i* ∈ *M*, then *l(i)* = 4, and if *i* ∈ *S*, then *l(i)* = 2, where *M* and *S* are sets of indices corresponding to proprioception and vision.

The TPM transformation is then described by the following formula:

(3) $$p_{i,t}=\frac{\exp\text{​}\left\{-{\frac{\left\|k_{i}\;-\;k^{\text{sample}}\right\|}{\sigma }}^{2}\right\}}{\sum_{j\;\in \text{ Z}}\exp\text{​}\left\{-{\frac{\left\|k_{j}\;-\;k^{\text{sample}}\right\|}{\sigma }}^{2}\right\}}$$

where in the case of proprioception (*i* ϵ *M*) then *Z* = 36 and *k*^sample^ = *m*_*t*_. While in the case of vision (*i* ϵ *M*) then: *Z*= 25, and *k*^sample^ = *s*_*t*_. σ is a constant set at 0.01, indicating the shape of the distribution of *p*_*i, t*_. Thus, *p*_*i, t*_ is a 61-dimensional vector transformed by the TPM to be the input to the MTRNN.

The MTRNN generates predictions of the next step sensory states based on the acquired forward dynamics described later. Similarly, outputs of the MTRNN were 61-dimensional vectors *y*_*i, t*_. The output of the MTRNN, assumed to correspond to an activation probability distribution over the TPM units, was again transformed into 6-dimensional vectors using the same TPM, as follows:

(4) $$k^{\text{out}}=\sum_{i\;\in \;Z}\;y_{i,\;t}k_{i}$$

where *Z* = 36, and *k*^out^ = *m*_*t* + 1_, in the case of proprioception. *Z* = 25 and *k*^out^ = *s*_*t* + 1_, in the case of vision.

#### Neurons activation in MTRNN

Neurons in the MTRNN are modeled according to a conventional firing rate model, in which the activity of each unit constitutes an average firing rate over groups of neurons. Continuous time characteristics of the model neurons are described by (1). Actual updating of *u*_*i, t*_ values is computed according to (5), which is a numerical approximation of (1).

(5) $$u_{i,\;t\;+\;1}=\left(1-\frac{1}{\tau_{i}}\right)u_{i,\;t}+\frac{1}{\tau_{i}}\left[\sum_{j\;\in \;N}w_{ij}x_{j,\;t}\right]$$

The activation of the *i*th unit at time *t* (*y*_*i, t*_) is determined by the following formula:

(6) $$y_{i,\;t}=\{\begin{array}{ll} \frac{\exp(u_{i,\;t})}{\sum_{j\;\in \;Z}\exp(u_{j,\;t})} & \text{if}\;i\in Z\\ f(u_{i,\;t}) & \text{otherwise} \end{array}$$

where *Z* is proprioceptive inputs. Note that the softmax activation is applied only to each group of output units, not to the context units. Activation values of the context units, however, are calculated according to a conventional sigmoid function *f*(*x*) = 1/1 + *e*^−*x*^. Application of softmax activation to the MTRNN makes it possible to maintain consistency with output of the TPM, which is calculated through use of the softmax function.

Activation values of the output units are then sent to the TPM and transformed into predictions of *m*_*t* + 1_ (the vision signal *s*_*t* + 1_ is not predictable at this stage since we designed the task, in this study, so that the interruptions and the switching signals occurred in an unpredictable manner). Based on *m*_*t* + 1_ prediction, the robot generates movement; as a result of this, actual sensory feedback ${\hat{m}}_{t\;+\;1}$ is sent to the system using the TPMs described earlier. Activation values of the non-output units *y*_*i, t*_, one the other hand, are simply copied as recurrent inputs to the neural states of the next time step, *x*_*i*_, _*t* + 1_.

### Training

In the proposed model, inputs to the system are the proprioception ${\hat{m}}_{t}$ and the vision sensor ${\hat{s}}_{t}$. Based on the current ${\hat{m}}_{t}$ and ${\hat{s}}_{t}$, the model generates predictions of proprioception *m*_*t* + 1_ for the next time step. The prediction of the proprioception is then sent to the robot in the form of target joint angles, which act as motor commands for the robot to generate movements and interact with the environment. For recording the initial teaching signals (the 16 combinations of task-A, task-B, and task-C), the experimenter guides the robot's right hand along the trajectory of each of the task sequences. The training of the network was conducted by means of supervised learning using these teaching sequences. The conventional back-propagation through time (BPTT) algorithm was used for learning of the model network (Rumelhart and McClelland, 1986). The objective of training was to find optimal values of connective weights minimizing sensory prediction error *E*, defined as the learning error between the teaching sequences and output sequences. In the current study, the BPTT was not used for mimicking the learning process of biological neural systems, but rather as a general learning rule.

At the beginning of training, synaptic weights of the network were set randomly (values ranging between −0.025 and 0.025). Connective weights approach their optimal levels through a process in which values are updated in a direction opposite to that of the gradient ∂*E*/∂*w*.

(7) $$w_{ij}(n+1)=w_{ij}(n)-\alpha \frac{\partial E}{\partial w_{ij}}$$

where α is the learning rate (constant fixed at 5.0 × 10^−4^) and *n* is an index representing the iteration step in the learning process. ∂*E*/∂*w* is given by:

(8) $$\frac{\partial E}{\partial w_{ij}}=\sum_{t}\frac{1}{\tau_{i}}\frac{\partial E}{\partial u_{i,\;t}}\;x_{j,t-1}$$

and is recursively calculated from the following formula:

(9) $$\frac{\partial E}{\partial u_{i,\;t}}=\{\begin{array}{ll} y_{i,\;t}-y_{i,\;t}^{*}+\left(1-\frac{1}{\tau_{i}}\right)\frac{\partial E}{\partial u_{i,\;t\;+\;1}} & i\in 0\\ \sum_{k\;\in \;N}\frac{\partial E}{\partial u_{k,\;t\;+\;1}}\left[\delta_{ik}\left(1-\frac{1}{\tau_{i}}\right)+\frac{1}{\tau_{k}}w_{ki}\;f^{\prime }\text{​}\left(u_{i,\;t}\right)\right] & i\notin 0 \end{array}$$

where *f'()* is the derivative of the sigmoidal function and δ_*ik*_ is Kronecker's delta (δ_*ik*_ = 1 if *i* = *k* and otherwise 0).

Through the calculations of the BPTT algorithm, the values of connective weights approach their optimal values by minimizing the error between teaching sequences and output sequences.

### Experiment

Ten learning trials were conducted with randomized initial synaptic weights for each type of network architecture (i.e., the two-level model and the three-level model). Successful trained weights were then tested through the interaction of the robot in the physical environment.

## Results

### A two-level model

**Figure 4:**
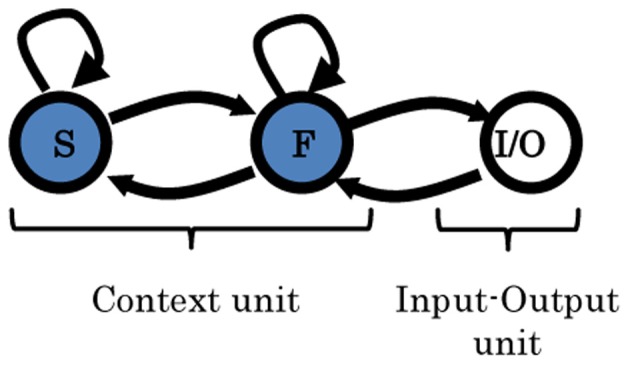
**Two-level MTRNN model.** Arrows indicate the direction of the synaptic connections between the units. The input/output unit (I/O) is fully connected to itself, as well as to the fast context unit (F). F-unit is connected to itself, as well as, to I/O and to the slow context unit (S). S-unit is connected to itself and to the F-unit as well. More details are given in Table 1.

As stated earlier, one of the targets of this work is to suggest a possible model structure that can self-organize a working memory capable of accomplishing cognitive branching and switching. In this experiment, we started with the minimum form of the MRTRNN structure, i.e., two-level context units, as shown in Figure 4.

**Figure 5:**
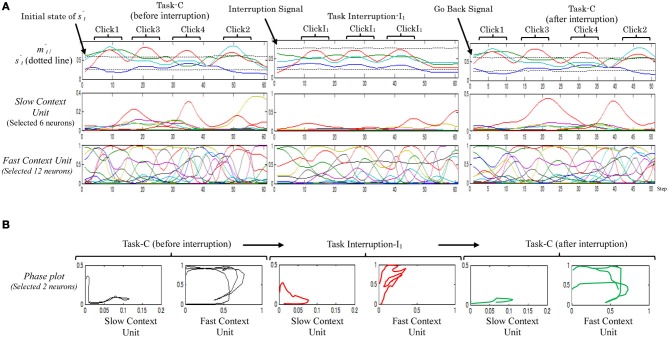
**A sample of the neural activities (A) and phase plots (B), resulting from the two-level Model while performing task-C with one set of task interruptions (Task-C → I_1_ → Task-C).** In the phase plot, red plots illustrate the task interruption part; black plots illustrate the original task before the task interruption; and green plots illustrate the original task after task interruption I_1_.

By considering this model, only one trial out of the ten trials successfully performed the desired task. Figure 5 shows an example of the neural activities of the fast and the slow context units of the successful trials when performing (Task-C → I_1_ → Task-C). From the figure, although the formulated memory in the slow context unit could maintain the original task to some limitation, i.e., the robot was able to return to the outstanding original task after performing the task interruption, it is obvious that if the interruption lasts for a little longer, the slow context unit will lose the ability to resume maintaining the memory. From the figure, the representations of the neural activations of task-C, before and after the task interruption are slightly different. Neurons in the slow context unit thus appear to be manipulated by the task interruption.

From these results it can be concluded that direct communication between the high- and low-unit can impair the ability of the working memory to preserve the original task since it will remain vulnerable to moment-to-moment environmental changes. Separation of the slow and fast context units, therefore, may solve this issue.

### A three-level model

**Figure 6:**
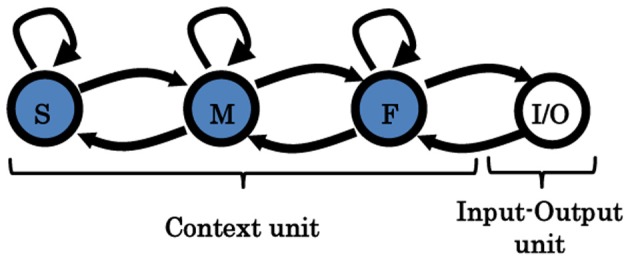
**Three-level MTRNN model.** Arrows indicate the direction of the synaptic connections between the units. In contrast to the two-level model, F and S are indirectly connected throughout the mid-context unit (M) to increase the distance between them.

Due to the limited performance of the two-level model, in this experiment, a three-level MTRNN model was constructed; see Figure 6. From the figure, the fast- and the slow-context units were separated from each other by introducing a mid-context unit. Re-conducting the 10 trials considering this model, 8 out of 10 learning trials could form proper working memories to successfully perform the desired tasks. From an analysis of the neural activities in the context units of the successful trials, interestingly, two types of working memory were observed: a static memory (five out of the eight) and dynamic memory (three out of the eight), wherein the neural dynamic representations of the slow context unit are significantly different.

#### Static memory

**Figure 7:**
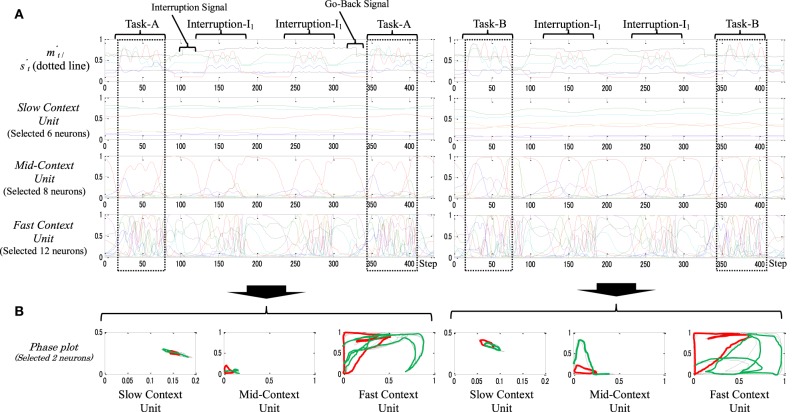
**(A)** An example of the behavior sequences and the neural activities of task-A [Task-A → I_1_ × 2 → Task-A (left)], and task-B [Task-B → I_1_ × 2 → Task-B (right)]. **(B)** Their phase plots. As is apparent in the figure, both tasks were subjected to two sets of a task interruption-I_1_. In the phase plot, red plots illustrate the task interruption stage; black plots illustrate the original task before the task interruption; green plots illustrate the original task after the task interruption; and the black and the green plots are almost identicals in the slow context unit.

Figure 7 shows an example of a static memory type model when performing task-A (left) and task-B (right). Both tasks were interrupted by two sets of interruption-I_1_. From the figures, the neural activities in the slow context unit seem to be generally stationary, i.e., neurons in the slow context unit were self-organized to form a role similar to that formed by the parametric bias (PB) (Yamashita and Tani, 2008). They represent, on a very abstract level, the selected original task (e.g., task-A, task-B) without any significant influence from the interruption task. The mid-context unit, in contrast, has been influenced, to a certain degree, by the ongoing task changes. Thus, marked changes appear between the representation of the original task and the interruption task. Finally, the fast-unit neurons seem to participate mainly in representing the primitives of the ongoing task (e.g., click-1, click-2, click-I_1_, etc.). Such neural representations can also be observed by the phase plot in Figure 7B; the plots show the various abstract levels in representing both tasks (from the general task goal at the higher level (slow context unit) to concrete motor responses at the lowest level (fast context unit).

Arrangement of such hierarchal neural representation helps the robot to obtain stability in holding the memory when dealing with multiple and/or long-term interruptions. Thus, from Figure 7B, the neural dynamics of the original task before and after the task interruptions is almost identical (see also the dotted squares in Figure 7A). We therefore call this type of memory a static memory.

#### Dynamic memory

**Figure 8:**
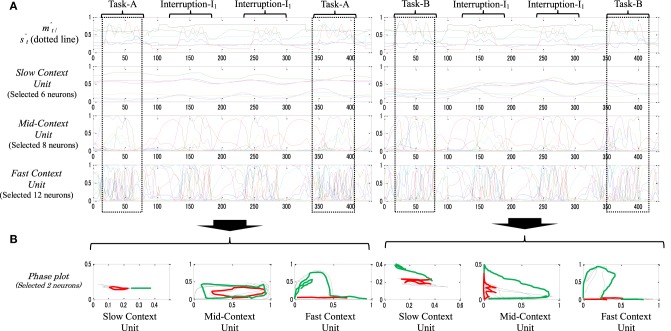
**(A)** An example of behavior sequences and neural activities of a dynamic-type memory while performing task-A [Task-A → I_1_ × 2 → Task-A (left)] and task-B [Task-A → I_1_ × 2 → Task-A (left)], with two sequences of the task interruptions-I_1_ B. Their phase plots **(B)**. In the phase plot, red plots illustrate task interruption part; black plots illustrate the original task before the task interruption; and green plots illustrate the original task after the task interruption. From the phase plots, particularly in the slow and mid-context units, slight differences between the representation of black and green plots are observed.

Figure 8 shows the neural activities of the context units of a dynamic memory type while performing task-A (left) and task-B (right), each with two sets of interruptions-I_1_. From the figure, in contrast with the static memory in Figure 7, the neural dynamics in the slow context unit here seem to be more influenced by the ongoing task changes. The neural representation of task-B, for instance, shifts slightly from its origin after the interruption tasks are introduced twice. This also can be observed significantly in the mid-context level (note the original task before and after the interruptions, Figure 8A dotted squares). The phase plots of Figure 8B confirm these observations; the phase plot of the slow and the mid-context units before (black) and after (green) the task interruptions are shifted relative to each other.

These results suggest that such a representation of the neural activities decreases the robot's stability when dealing with a longer period of interruption. Thus, the neural representations that are used to suspend (encode) the original task, task-B for instance, will gradually fade after a few repetitions of the interrupting subtask, and thus returning to this task becomes impossible. We call this type of memory dynamic memory.

### Static vs. dynamic memory

To better understand the reason behind of the emergence of these two types of memories and the resulting characteristics of each, we have investigated (1) the contribution of each context unit in each type of memory in encoding the original task; and (2) the connectivity between the context units in each type of memory.

#### Contribution of the context units

**Figure 9:**
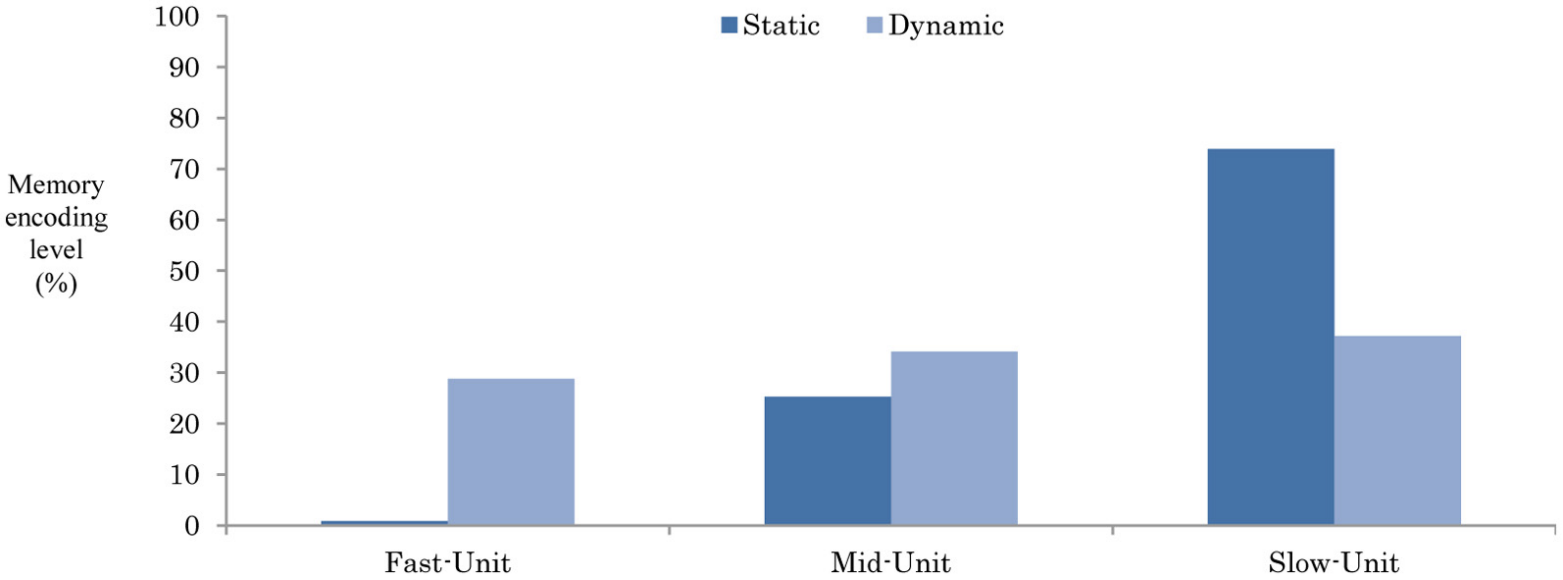
**The encoding level of the original tasks while responding to the task interruption-I_1_ (10).** The plotted data are the average of neural dynamics of each unit for an average of the three static and dynamic working memories.

To obtain insight into the functionality acquired at each context unit in each memory type, as well as the contribution of its neurons to encode the original task in the branching process, we have investigated each unit's neural dynamics while suspending the original task of responding to the interruption task. The idea here is that if the same task interruption I_1_, for instance, is introduced to two original tasks, Task-A, and Task-B, e.g. (Task-A → I_1,*A*_ → Task-A), (Task-B → I_1,*B*_ → Task-B), then the difference *d* between the neural activities within the interruption period (i.e., I_1,*A*_ – I_1,*B*_) will reflect the contribution degree of the unit in encoding the memory. Context units that represent higher distances will be considered as units that are highly involved in encoding the original task, and *vice versa*. Since in this study we deal with 3 original tasks, Task-A, Task-B, and Task-C, *d*_*i*_ is calculated by Equation (10).

(10) $$d_{i}\text{​}=\text{​}\frac{\frac{\sum_{t\;=\;0}^{T}|x_{t,\;A}-y_{t,\;B}|}{T}\text{​}+\text{​}\frac{\sum_{t\;=\;0}^{T}|x_{t,\;A}-y_{t,\;C}|}{T}\text{​}+\text{​}\frac{\sum_{t\;=\;0}^{T}|x_{t,\;B}-y_{t,\;C}|}{T}}{3}$$

where *i* represents the neuron in the context units (Table 1). *x* and *y* represent the neural activity of task-A and task-B, task-A and task-C, and task-B and task-C, respectively. *T* represents the total steps of the task interruption (T = 60 steps). Figure 9 shows the normalized average of the distance of the neurons in each unit to 100% (i.e., the summation of elements in each unit equal to 100).

From Figure 9, in the static-type memory, the original task seems to be highly encoded into the slow context unit (73.9%), and partially aided by the mid-context level (25.3%). Recruiting the neurons in such as structure appear to increase the distance between the slow-unit and the fast-unit, which therefore preserves the working memory from any instant moment-to-moment changes in the environment. In contrast, encoding the memory in the dynamic memory appears to be mainly shared between the slow-, mid-, and the fast-units (37.1, 34.1, and 28.8%, respectively). Such encoding decreases the distance from the memory to the environment, and thus impairs the memory ability when dealing with long-term interruptions.

#### Connectivity among the context units

**Figure 10:**
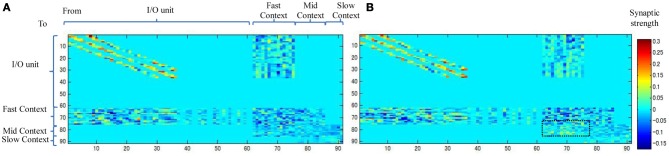
**Synaptic weight matrices for (A) Static memory and (B) Dynamic memory (the plots are the average of three static and dynamic memories).** Synaptic connections between units are shown in Figure 6.

Figure 10 shows the weight connectivity matrices resulting from an average of the three static and dynamic memories. From the figure, we observe that the dynamic memory tends to have a dense connectivity from the fast to the mid-context units than the static memory (the dotted square in Figure 10B). A dense bottom-up synaptic connectivity between the fast and the mid-context units appears to reduce the distance and facilitate the transmission of the dynamic property of the neurons from the fast context unit to the slow context unit. This, accordingly, reduces the stability of the memory and leads to dynamic memory, and *vice versa*.

From these results, we can conclude that the distance between the slow context unit and the fast context unit is based entirely on the initialized random weights, which play a key role in the formation of the characteristics of the resulting memories. One interesting future research direction related to these results would be to investigate the possibility of changing the properties of the resulting working memories by subjecting them to certain exercises (e.g., repeating the original task for a longer period before introducing the interruption). This issue is scheduled for future study.

### Task switching

To examine the flexibility between the resulting static and the dynamic memories, both types of memories were introduced to the task switching. This stage was conducted in the simulation environment only. After a number of repetitions of the original task, we asked the robot to switch to another original task. This was made possible by changing the value of ${\hat{s}}_{t}$ to one of the switching positions; see Figure 3. This step was repeated for 10 trials.

**Figure 11:**
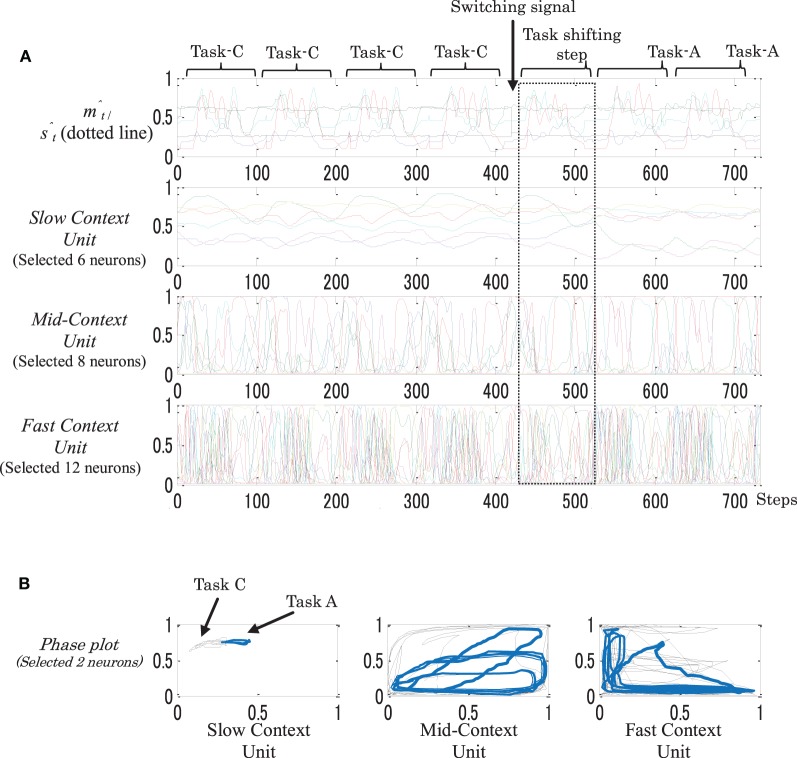
**An example of behavior sequences and neural activities (A), and phase plots (B) of a dynamic memory while performing task switching from task-C to task-A.** In the phase plots: black plots illustrate task-C and blue plots illustrate task-A. After introducing the switching signal, a gradual shift appeared on the neural activations in all the context units, starting from the fast context unit and moving to the slow context unit.

For the dynamic memory, task switching was successfully achieved among all the trials. This reflects the high flexibility of this type of memory. For the static-type memory, on the other hand, in all the trials the network was unable to switch between one or two tasks. This could be due to the high stability of the higher context unit, which was only very slightly affected by moment-to-moment changes on the environment. Figure 11 shows an example of the neural activities of a dynamic memory while successfully performing task switching from Task-C to Task-A.

From the figure, when the ${\hat{s}}_{t}$ value was changed to stimulate the task switching S_1_ (switch to Task-A), as seen in Figure 3, a gradual shift of the neural activations appeared in all the context units, starting from the fast context unit and moving to the slow context unit (dotted square). As the result, the network was successfully able to switch to Task-A.

In conclusion, dynamic memory has greater flexibility for moment-to-moment changes in the environment than does static memory.

## Discussions

In this study, we proposed a neurocomputational model that may suggest the basic perception of how a working memory in human brain may be mechanistically self-structured through neural interactions to accomplish HOCM, namely cognitive branching and switching.

First, a minimum form of the MRTRNN structure was examined (a two-level MTRNN model). The model poorly performed cognitive branching (only one out of 10 trials succeeded). Although the model could cope with segmentation and integration of the introduced tasks, it was hardly able to self-organize a suitable working memory to handle its pending stages. The problem could lie in the distances between the context units, which seems to have a significant impact on the formulation of the resulting working memory.

Second, we proposed a three-level MTRNN model, which outperformed the two-level model. The branching tasks were successfully performed in 8 out of 10 trials. The results illustrate the importance of a separation between the slow-unit (high-level) and the fast-unit (low-level) for the formation of a proper working memory away from any lower-level processors, sensory noise, and/or moment-to-moment changes in the environment. These results are remarkably consistent with previous findings, including the “bottle-neck” in Paine's work (Paine and Tani, 2005), where neurons of the higher module, which carry information about the task goal, interact with external inputs of the lower module only through a particular class of neurons referred to as bottle-neck neurons. This ensures a kind of separation between the higher level and the lower level. Botvinick's work in Botvinick (2008) also suggested that a separation from input-output unit can contribute to the development of a functional hierarchy.

By further analysis of the successful trials, interestingly, we observed that two types of memory, static memory or dynamic memory, were self-organized during training. The static memory appeared in five out of the eight trials. Static memory showed high stability in preserving the original task and less flexibility with moment changes on the environment. Thus, it carried out the cognitive branching task better than the switching task. In this type of memory, the neural dynamics in the slow context unit was mainly engaged in encoding the memory, playing a role similar to that of a PB (Yamashita and Tani, 2008). The dynamic memory appeared in three out of eight trials. Dynamic memory showed low stability to maintain the original task and more flexibility to the ongoing environmental changes. It thus better performed switching compared to branching. In this type of memory, the slow-, mid-, and fast-context units seemed to be all involved, to various degrees, in encoding the memory. Surprisingly, relatively comparable types of memory representations were also found a similar investigation of other higher-order cognitive tasks, namely, rule switching and confidence (Maniadakisa et al., 2012).

From an analysis of the neural activations in both types of memory, we believe that the variances of the strength between bottom-up and top-down synaptic connections, which were randomly initialized and self-organized throughout the learning process, are the main factor to form the properties of the resulting memories. Higher bottom-up weights, which could facilitate the transmission of information into that direction, reduce the distance between the lower and the higher units, and thus lead to dynamic memory. Higher top-down synaptic weights, in contrast, can reduce the impact of any lower-level processors, and thus increase the distance between the lower and the higher units, which lead to a static memory. Optimal balancing between these distances, therefore, could serve as a basis to attain balance among those memory types: this phenomenon which may reveal some interesting characteristics of the frontal lobe to handle HOCM. This may account for our daily psychological observation that some people are able to more effortlessly perform cognitive branching and switching than others. There is also a possibility that both types of memory may also emerge in one model. This can happen if there is a certain balance rate between the units in the model. In this case, an extra variable synaptic weight, which is less dependent to the training process and more dependent on the environmental changes, can perhaps be used to convert between one type of memory and the other, depending on the situation. This also could be linked to the term “mood” in human, where in the case of a good mood we have greater ability to perform cognitive branching than when we are in a bad mood (Walsh et al., 2009).

To some degree, our resulting model and the review of frontal lobe studies in Badre and D'Esposito (2009) (mainly by looking at the layout structure and task development) can be associated: where the fast-unit can be assigned to the lateral FPC, the mid-unit to the Mid-PFC, the fast-unit to the premotor cortex, and the input-output-unit to the posterior sensory-motor cortex); see Figures 1, 2. It is important to note here that, although the connectivity between the units in the proposed three-level MTRNN model is not fully compatible with the frontal lobe connectivity assumed from neuroimaging studies discussed in Badre and D'Esposito (2009), we believe that it does not conflict with it. Thus, their reported direct connection from the high-level unit (FPC) to the low-level unit (premotor cortex) could possibly be critical for the emergence of other human higher cognitive abilities (e.g., complex decision making, etc.) rather than the selected tasks in this study. Another possibility could be that this extra link could work as an additional factor to balance the memory to cope with various situations.

A complete frontal lope model should host more complex functions for higher-order cognition, such as novelty and unexpected choices, online configuration of old memories, etc. However, the current paper focuses only on specific mechanisms related to cognitive branching and switching. It is important to integrate those various higher functions in future studies which would require more complex architectures than the current simple architecture.

## Conclusions

In the present work, we conducted neurorobotic experiments driven by a neurocomputational model to suggest a possible neural dynamics for ruling cognitive branching and switching in human brain. Our results suggest a possible network structure and neural dynamics involved in accomplishing tasks.

Interestingly, two types of network emerged from training the proposed three-level MTRNN model. Each of these networks has its own memory characteristics: either static memory or dynamic memory. The existence of any of these memories mainly depends on the resulting distances from a long-term learning between the contexts units that involved in the process. Both memories could successfully perform the desired tasks with some limitations; the static type memory performed well in terms of stability, but showed poor flexibility, in dealing with the tasks. The dynamic type memory showed converse performance.

From the results, we believe that extension of this work could contribute to possible neural implementation for better insight into how macro-level anatomical nodes in the frontal lobe are dynamically structured and self-organized to obtain various HOCM. An important issue for future work will be to scale both the model and the task into further complex levels in which the formed memory should not only encode the main ongoing task as a set but also encode to which of the task sequence steps the interruption occurred. The limitations of the biological plausibility of the resulting model are also a subject for detailed explorations in the future.

### Conflict of interest statement

The authors declare that the research was conducted in the absence of any commercial or financial relationships that could be construed as a potential conflict of interest.
